# Return(s) on investment: Restoration spending in the Columbia River Basin and increased abundance of salmon and steelhead

**DOI:** 10.1371/journal.pone.0289246

**Published:** 2023-07-28

**Authors:** William K. Jaeger, Mark D. Scheuerell

**Affiliations:** 1 Department of Applied Economics, Oregon State University, Corvallis, OR, United States of America; 2 U.S. Geological Survey Washington Cooperative Fish and Wildlife Research Unit, School of Aquatic and Fishery Sciences, University of Washington, Seattle, WA, United States of America; University of Idaho, UNITED STATES

## Abstract

The decline in salmon and steelhead populations in the Columbia River Basin has been well documented, as have the decades-long, $9 billion restoration spending efforts by federal and state agencies. These efforts are mainly tied to Endangered Species Act (ESA) mandates for recovery of wild, naturally-spawning threatened or endangered fish species. The impact of these efforts remains poorly understood; many observers, including the federal courts, have long been concerned by the lack of evidence of recovery. Most studies evaluating restoration efforts have examined individual projects for specific species, reaches, or life stages, which limits the ability to make broad inferences at the basin level. There is a need to ask: is there evidence of an overall increase in wild fish abundance associated with the totality of these recovery efforts? To that end, the current study estimates fixed-effects panel regression models of adult returns of four species. Results indicate that restoration spending combined with hatchery production are associated with substantial increases in returning adult fish. Evidence of benefits to wild fish alone, however, require indirect approaches given the commingling of restoration spending with spending on hatchery releases, the impacts of spending on hatchery fish survival, and the density dependence effects of hatchery releases. To accomplish this, the models’ predicted adult returns (both hatchery and wild fish) attributed to both spending and hatchery releases are compared to independent estimates of returning hatchery fish based on hatchery survival estimates (smolt-to-adult ratios). The comparison finds the model-predicted levels of adult returns due to spending and hatchery releases do not exceed the survival-rate based estimates for hatcheries alone, so that we are unable to reject the hypothesis of no benefits to wild fish from the restoration spending.

## Introduction

**Fig 1:**
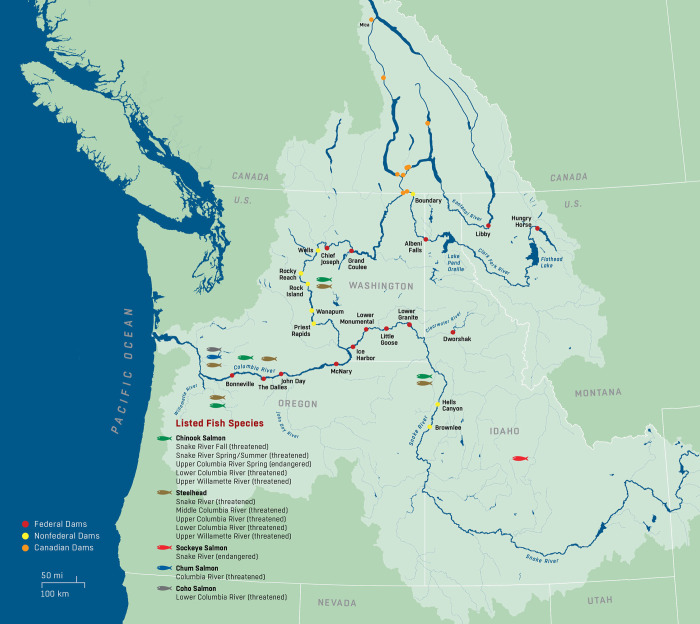
Map of the Columbia River Basin.

The decline in populations of salmon and steelhead in the Columbia River Basin (CRB) since the second half of the 19^th^ century has been well documented [1, 2], as have the ensuing decades-long, multi-billion dollar efforts toward their restoration and recovery [3, 4]. Historical adult populations migrating above Bonneville Lock and Dam (Bonneville Dam hereafter), between states of Oregon and Washington, USA, estimated at between 5 and 16 million, declined by the 1970s and 1980s to less than one million fish (www.fpc.org/). These declines resulted in part from decades of overharvesting from the 1880s to the 1920s (estimated to have caught 85% of all returning adult fish [5]), and subsequently in the 1930s to 1971 by construction of dams on the mainstem of the Columbia River, blocking or impeding juvenile fish from accessing their freshwater spawning grounds (Fig 1). Over the same period, farming, logging, mining and irrigation contributed to landscape changes and degradation, reductions in riparian vegetation and deforestation: watercourses were blocked by culverts, diversions, and private dams, which added to the many factors adversely affecting these salmon and steelhead populations [1].

Pacific salmon have a unique life cycle that exposes them to a variety of natural and anthropogenic forces [6]. During the summer and fall these anadromous fish lay their eggs in nests, or redds, created by females in the bottom of streams, rivers, and lakes, where the eggs develop over the winter before hatching the following spring. Apart from steelhead (*Oncorhynchus mykiss*), which can spawn more than once, adult Pacific salmon die shortly after spawning. Juvenile salmon then rear in freshwater ecosystems for anywhere from a few weeks to a few years before they migrate–often covering distances of hundreds of miles–to the ocean where they forage and grow for generally one to four years before maturing and migrating back into estuaries and ultimately freshwater to spawn.

The tenuous plight, ongoing recovery efforts, and debate about restoration of Columbia River salmon and steelhead have been a major concern for scientists, local communities, fishers, tribes, environmentalists, resource managers, policymakers and politicians for many decades [1]. Early programs undertaken to augment Columbia River salmon and steelhead populations followed passage in Congress of the 1938 Mitchell Act, which promoted artificial production in hatcheries to compensate for habitat lost as a result of the construction of dams that blocked access or impeded fish passage to large portions of the upper Columbia River and Snake River basins. More broad-based restoration programs in the basin followed passage of the 1980 Pacific Northwest Electric Power Planning and Conservation Act (Northwest Power Act), which required consideration of fish and wildlife objectives alongside those of power generation and other goals. This law mandated creation of the Northwest Power and Conservation Council (renamed from its early title) which established their Fish and Wildlife Programs financed by revenues from power generation by the Bonneville Power Administration (BPA). Also notable was the U.S. Fish and Wildlife Service’s (USFWS) Lower Snake River Compensation Plan authorized by Congress as part of the Water Resources Development Act of 1976. The Plan was intended to mitigate for the loss of fishery resources caused by construction and operation of the four lower Snake River hydroelectric dams. The resulting program currently operates 11 hatchery facilities intended to produce enough smolts to return 132,000 adult salmon and steelhead annually (https://www.fws.gov/pacific/fisheries/Documents/LSRCP%20Fact%20Sheet%202015.pdf).

Following the listings in the 1990s of 12 Columbia River runs of salmon and steelhead as threatened or endangered under the Endangered Species Act (ESA), the scale and cost of restoration efforts grew significantly to become among the largest in the world. More than 9 billion USD ($2020) has been spent over the past four decades on restoration efforts by federal and state agencies, along with additional actions by local governments and non-governmental actors [7, 8]. Other analyses cite considerably higher spending amounts for CRB conservation programs. These higher tallies reflect BPA cost estimates that include spending on other wildlife and resident fish programs, and also include both estimates of BPA’s ‘foregone revenues’ (the opportunity cost when water is spilled, bypassing power-generating turbines, and thus generates no power revenues), as well as “power purchases” (“when fish operations cause BPA to purchase power to meet its load obligations, the cost of purchased power is identified as a fish cost”).

In recent years, off-site measures (not directly related to dam operations) have been a major focus of efforts to mitigate for the impacts of mainstem dams on fish mortality. In particular, habitat restoration and improvement has been prioritized as a way to improve salmon habitat and potentially increase salmon numbers to meet recovery goals.

Many of these restoration projects, however, are funded on an *ad hoc* basis depending on the priorities of the funding agencies, availability of funds, political equity considerations, and ease of implementation rather than specific needs outlined in recovery plans [4, 9]. These kinds of political equity concerns may actually result in minimizing the benefits to society (Wu et al. 2003). For projects in the Columbia Basin’s Fish & Wildlife Program, evaluating the return on these investments is complicated by conflicting objectives and the scales at which they are applied [10]. Moreover, prioritizing of Fish and Wildlife Program projects on the basis of cost-effectiveness criteria, mandated under the 1980 Northwest Power Act, has not been carried out [11].

The aim of recovery efforts mandated under the ESA for salmon and steelhead in the Columbia River Basin is the restoration of wild, naturally-spawning fish populations listed as threatened or endangered. Those recovery efforts involve eleven federal agencies. To achieve that goal, the Federal Columbia River Project System (FCRPS) Biological Opinion (BiOp) requires the federal agencies (Army Corps of Engineers (USACE), BPA, and Bureau of Reclamation (BOR), etc.) to implement a series of mitigation measures [12]. The lead responsibility is held by the National Oceanic and Atmospheric Administration National Marine Fisheries Service (NMFS) for preparing recovery plans in consultation with other agencies to determine whether their collective planned actions will jeopardize the ESA-listed populations of fish. For its part, the Bonneville Power Administration is responsible for providing power transmission and marketing services for electric power generated by the USACE and BOR dams in the CRB. Under the 1980 Northwest Power Act, however, the BPA is also obligated to provide equitable treatment to fish and wildlife along with the other purposes of the hydropower system; and the Northwest Power and Conservation Council is responsible for supporting conservation and efficiency, for example in determining whether actions being employed are cost-effective [13].

The ESA requires federal operators of the CRB hydropower system to consult with the NMFS on how its operations may impact listed species. At the end of such consultations, the NMFS issues a BiOp indicating whether the hydro-system operations would jeopardize the future existence of a listed species or damage its critical habitat [2]. If jeopardy is found, NMFS is required to develop a program of “reasonable and prudent alternatives” in order to avoid jeopardy.

**Fig 2:**
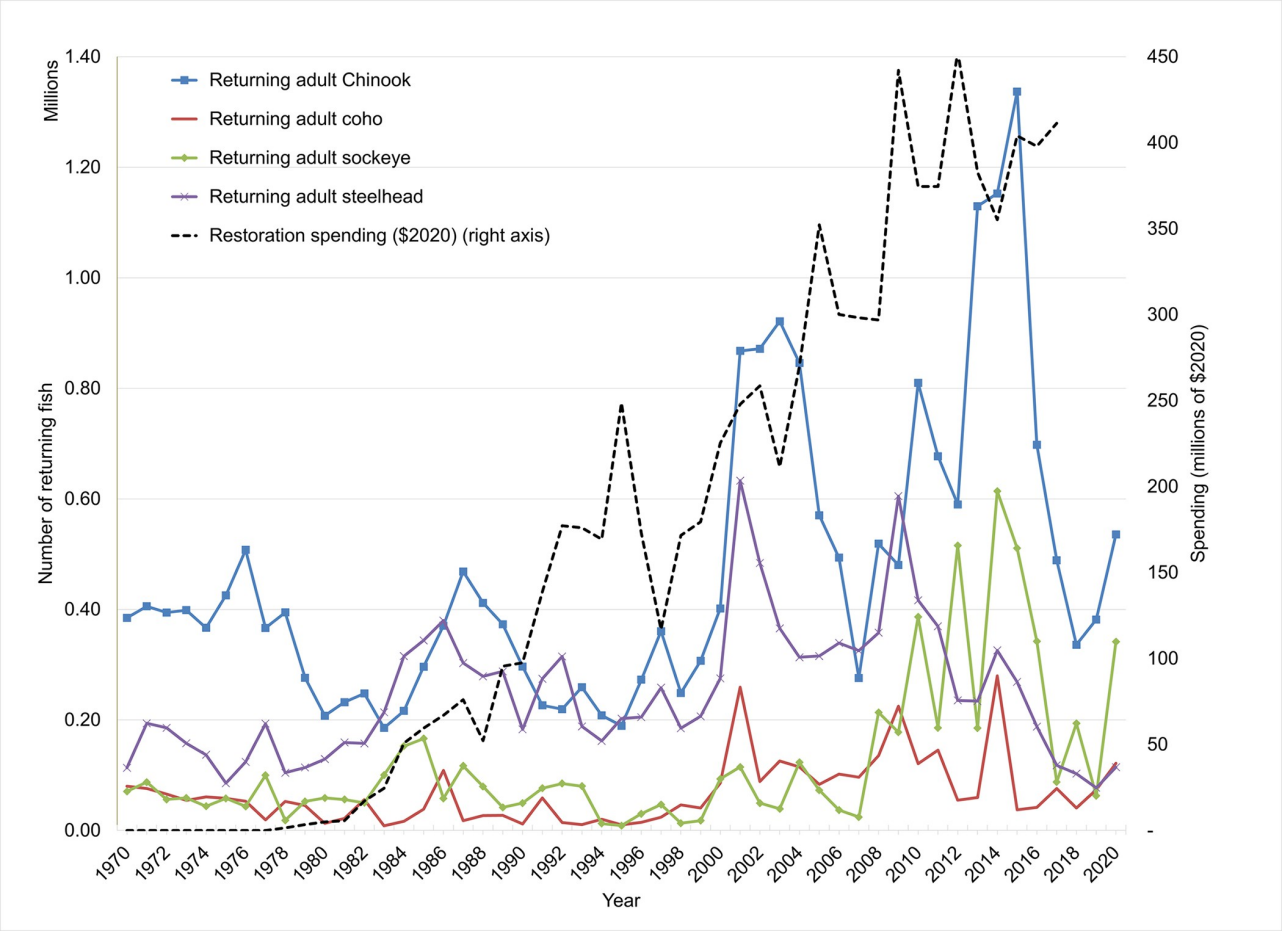
Adult returns at Bonneville Dam & restoration spending over time.

Despite several decades of federal agency actions in response to these requirements, many observers including local and state governments, community groups, and stakeholders, have been stymied by the paucity of evidence of improvements in fish populations despite these actions and high levels of expenditures [14, 15]. Although the Northwest Power and Conservation Council set a quantitative goal of increasing total salmon and steelhead abundance to 5 million fish by 2025 for the basin as a whole [16], adult returns at Bonneville dam averaged less than 1.5 million in the 2010s based on Fish Passage Center data (see Fig 2 and www.fpc.org).

Beginning in 1992, NMFS has issued a sequence of BiOps for ESA-listed fish in the CRB, nearly all of which have been found to be noncompliant with the ESA. Since 2000, all or parts of multiple BiOps and their supplements have been rejected by the courts, including finding the 2014 Supplemental BiOp to be arbitrary and capricious [2]. The assemblage of these federal recovery programs has repeatedly been rejected by the US courts as failing to be in compliance with the requirements of the ESA, mainly for failing to identify or document specific mitigation measures and plans that were reasonably specific or reasonably certain to occur. In part, little evidence could be shown that the measures were working or were promising as reasonable and prudent actions that would not jeopardize any of the listed species’ likelihood of recovery. The courts have concluded, in 2016 for example, that there is a lack of evidence to support the claim that the species were “trending toward recovery.” (National Wildlife Federation et al. v. National Marine Fisheries Service, No. 3:01-cv-00640). The most recent 2020 NMFS BiOp [17] describes a set of final proposed actions, ones similar to those previously rejected, but concluding nevertheless that they will not likely jeopardize the continued existence of the ESA-listed species.

**Fig 3:**
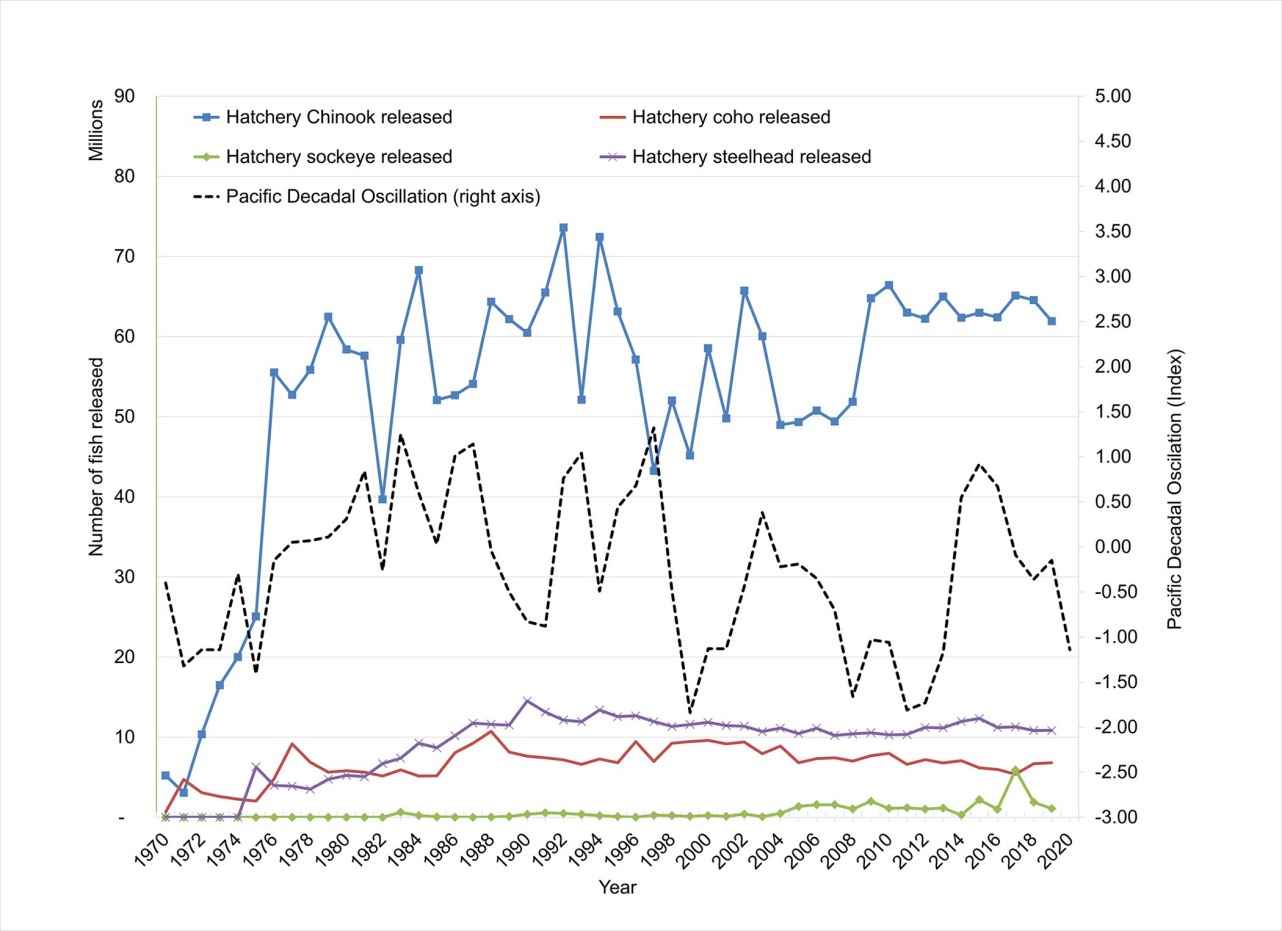
Hatchery releases and Pacific Decadal Oscillation over time.

One critical element for salmon and steelhead in the CRB is the role of artificial production. Hatchery production began in the 1880s to support cannery operations and prevent the need for harvest regulation [18] Hatchery production grew to high levels following the Mitchell Act of 1930 and subsequent additional hatchery development by federal, state, and tribal actors. Hatchery released juveniles currently include those from 82 federal, state and tribal hatcheries, amounting to approximately 140 million salmon and steelhead released annually [19]. Importantly, the ESA listings and subsequent BiOps in the CRB include hatchery production, seemingly counterintuitively, as part of ESA-mandated recovery plans to restore naturally-spawning, or wild fish. Although the aim of the ESA mandate is the restoration of wild, naturally-spawning fish populations listed as threatened or endangered, hatchery production is included as part of these efforts, being promoted as a tool to help support wild stocks and to provide fish for harvest. Survival of both naturally-spawning and hatchery released fish are vulnerable to variations in ocean conditions, indicated for our purposes by Pacific Decadal Oscillation or PDO (see Fig 3), but with the proviso that hatchery-reared fish be managed in the context of the recovery goals for the ESA-listed (wild) fish. The role of hatchery production in these recovery plans is controversial for several reasons. Hatchery production has increased numbers of adult fish, but has also negatively impacted wild stocks through a variety of mechanisms including competition for habitat, food supply, genetic effects and disease, predation by hatchery fish on wild fish, and other adverse effects [20, 21]. In addition, larger populations of returning hatchery salmon and steelhead can put added pressure on fishery managers to allow higher catch limits in these mixed-stock fisheries for commercial, recreational and tribal fisheries for which selective harvest of only hatchery fish cannot be fully ensured [22].

Most studies evaluating the impacts of these CRB restoration programs have looked at individual projects for specific species, reaches, or life stages, and as a result they can at best provide only limited evidence of recovery at the basin level. A central question is: what evidence is there of an overall increase in wild fish abundance associated with the totality of these recovery efforts? The CRB situation is ripe for a more aggregate empirical approach, in part due to the extraordinary basin-wide data available covering 50 years for adult returns, hatchery releases, restoration spending, and other environmental factors.

Our approach addresses this central question by juxtaposing two complementary analyses. The first of these is our empirical regression analysis of the relationship between restoration spending in the CRB, other explanatory variables, and the levels of returning adult salmon and steelhead–both wild and hatchery produced. The second analysis computes an independent estimate of hatchery adult returns based on estimated survival rates and numbers of released fish. The hypothesis that restoration spending has benefited wild fish abundance is tested by comparing these two analyses.

## Restoration and recovery expenditures in the CRB

The programs and expenditures aimed at restoring CRB salmon and steelhead cover a wide range of activities that have been implemented by a variety of actors. The participants include federal agencies and state and local governments, tribes and NGOs. In the past 40 years ending in 2017 for which data are available [7], federal expenditures for ESA-related actions were 8.2 billion ($2020) (see Fig 2). Of this, 43% was for BPA programs and 27% for USACE. National Oceanic and Atmospheric Administration (NOAA) programs accounted for 12%, followed by BOR with 6% and USFWS with 3%. During that time period spending under BPA and USACE programs has grown and has been higher ($100–150 million/year) than for other federal agencies whose ESA-targeted spending has remained at lower levels (<$50 million/year).

Among the categories of spending over the past 20 years, the largest share of BPA spending was 36% for research, monitoring, and evaluation (RM&E), followed by 24% for habitat protection and restoration, 21% for planning and coordination, and 14% for hatcheries. Of these activities, many are intended to address restoration of one population of a given listed species. For example, BPA expenditures on ESA-listed species have risen to high levels over the past 20 years for Chinook and steelhead restoration programs ($80 million per year), whereas spending on ESA-listed Sockeye has remained at $5 to $10 million/year over the same time period. For the USACE, spending has similarly been higher for Chinook and Steelhead projects and lower for Sockeye.

The attribution of spending to a particular species may, in some cases, be more reflective of accounting conventions than of their potential impact. Programs aimed at improved habitat, increased dam passage survival, and reduced predation will benefit multiple species despite being targeted to one species. Similarly, hatchery expenditures include capital, operations and maintenance (see https://www.cbfish.org/Report.mvc/AllReports), are likely to be beneficial to multiple species since many hatcheries produce more than one species (for example, the Little White Salmon National Fish Hatchery produces fall and spring Chinook salmon as well as coho salmon; the Eagle Creek National Fish Hatchery produces steelhead and coho), and also because funding allocations are likely fungible between species-specific operations.

In the early years following the passage of the 1980 Northwest Power Act, the Northwest Power and Conservation Council’s Fish and Wildlife Program included efforts to benefit fish survival that did not amount to direct expenditures. Among those early actions was the establishment of a “water budget,” an amount of water reserved in upriver storage reservoirs in winter that would subsequently be released to increase downstream flows and aid the migration of juvenile salmon and steelhead to the ocean [23]. The size of the water budget was increased from 3.45 million acre-feet (MAF) to 12 MAF during the 1980s and 1990s. Additional flow and spill management actions have been introduced, up to the present time, in continued efforts to improve juvenile fish survival. Water requests have increased from 3.75 MAF in 1983 when the water budget was established to between 13 and 16 MAF in the 1995 and 2000 NMFS BiOp [24, 25]. For example, the BOR provided flow augmentation volumes for salmon of between 90,000 and 437,000 acre-feet between 1991 and 2004 [26].

## Evaluating the impacts of restoration expenditures

The effectiveness of these restoration programs remains poorly understood, and yet is critical to policy, management, investment, and legal decisions. One of the empirical challenges for measuring impacts of restoration actions in the CRB is the scale and scope of the system, its components, and their linkages. Efforts to evaluate the effects of CRB recovery programs have tended to examine individual actions aimed at specific species, life stages, tributaries, or even stream reaches. These include assessments of habitat improvements impacts on numbers of juvenile fish (spawners or smolts); survival rates for modifications in dam spill, passage, or transport; changes in hatchery practices including release timing or location; and interventions to cull avian predators. One drawback of these approaches is that they tend to address only one limiting factor or source of mortality for one stage of the life-cycle of a given population. However, it is well-known that relaxing or removing a limiting factor on growth or survival in one life-stage may not result in changes in abundance for the entire life-cycle if a) the limiting factor for the life-cycle overall exists in a different life-stage, or b) if improvements in one life-stage lead to compensatory or endogenous reductions in survival at another life-stage [27, 28].

Uncertainties include possible compensatory effects and general density-dependent survival (density dependence refers to the way that a change in the numbers or density of fish in a particular area cause a change in their population growth, most commonly a slowing of population growth due to high density). Given these effects, there is a strong rationale for a more aggregated approach to ask: what evidence is there of an overall increase in fish abundance associated with the totality of recovery efforts intended to promote recovery and restoration of CRB salmon and steelhead? For measuring the outcome in terms of abundance of fish, we are able to take advantage of a unique aggregate measure in the CRB for the number of adult salmon and steelhead returning at Bonneville Dam; recorded numbers of returning fish that have been counted continuously for more than 40 years at a single confined entry point to a freshwater habitat the size of France. This fact lends itself to a unique opportunity to examine these relationships for four species and all the CRB that lies upstream of the first or lowermost dam in the basin, the Bonneville Dam.

On the other side of the relationship, we want to estimate the potential impact of the suite of programs, project and interventions taken to promote salmon and steelhead recovery. Given the temporal range of expected impacts, life-stages and species affected, and wide range of actions intended to benefit one versus multiple species, a second empirical challenge involves assembling efforts into categories or a weighting scheme that would be complicated and raise difficult indexing or weighting issues. Aggregations of this kind are standard practice in economics, however, where the cost of inputs into a production process are added based on their cost or value. Economics of production at the farm, firm, corporation, or even the national level often involve assessing changes in output in relation to changes in various inputs, investments, labor, land, or equipment, all measured in monetary terms. Labor productivity, for example, is a regularly reported and useful statistic that measures output per hour of labor input where the outputs are measured in monetary units all the while including a multitude of types of goods and services.

Actions taken to restore salmon and steelhead populations in the CRB are exceptionally varied by type, location, geographic scale, durability, and life-stage affected. Aggregating recovery expenditures provides an imperfect but direct and intuitive way to measure the level of recovery effort. One can interpret the relationship as involving investments in the “capital” of the CRB, and we are interested in evidence about the return on those investments. Approaching conservation planning in this way is not uncommon for measuring the benefits resulting from a set of investments over a period of time [29–31]. This is generally referred to as “return on investment” (ROI) analysis. More generally the relationship between attributes of an ecosystem and the ecosystem services it generates can be represented empirically where conservation investments are translated into outcomes such as improved water quality or increased fish abundance. Such relationships are often referred to as “ecological production functions.” There are empirical studies of these relationships for various ecosystem services [29]. For instance, there is evidence that expenditures by environmental groups improve local water quality [32]. There are also several studies that specifically find evidence of positive impacts of government expenditures on endangered species recovery [33–35]. However, most of these studies do not include salmon or steelhead in their analysis.

The standard rationale for aggregating the costs for a wide range of diverse program categories relies on the implicit assumption that investors are making efficient decisions across all inputs or actions. If that were the case–and presumably many conservation programs endeavor in this way to get the most out of their limited budgets–we would expect each type of expenditure to be taken up to the point where the marginal return per dollar was equal across all types of investments. In the current context (where we cannot assume cost-effectiveness), an efficient mix of natural capital (habitat and environmental factors) and investments in habitat improvements, spill and bypass activities, research, monitoring and evaluation, control of predator, regulations on farming, forestry, and other land use activities, would over time produce an optimal improvement in the levels and resilience of fish populations.

Although we cannot assume in the current context that expenditures are allocated efficiently, it is reasonable to assume that some components of the mix of recovery actions complement each other and, in the aggregate, produce impacts that should be empirically associable with dollar expenditures. These expenditures include both short-term spending (e.g., hatchery releases) and long-term durable investments (e.g., habitat restoration), and these cannot be separated to test their individual influence (in fact, they cannot be separated since they are interdependent, complementary, and their productivity may not be separable in a real sense). Their aggregated combined influences can nevertheless be explored by testing a range of assumed durations of impacts or payoffs.

An additional feature of the data, one that strengthens the advantage of a long time series of adult return counts at Bonneville Dam, is having separate counts of adult returns for the four species of salmon and steelhead at issue for these restoration programs (the data do not distinguish wild from hatchery-reared fish however). This makes it possible to treat the four species as a panel data set (longitudinal data with observations for the same subjects–in this case species–in each time period). This approach adds considerable statistical power due to increased sample size and varied life cycle lengths and thus species-specific temporal influences (e.g., varied life-stage influences for ocean conditions, hatchery releases, etc.). As a result, these data and the empirical approaches described below have arguably the best chance to estimate the total impacts of restoration spending on fish abundance in the CRB.

## Empirical approach

To predict the overall increase in wild fish abundance associated with these recovery efforts, we first identify the combined effects for both restoration spending and hatchery production on wild and hatchery returning adult salmon and steelhead. We do this because of confounding factors and interactions between conservation spending and hatchery releases. First, the data on restoration spending include substantial levels of spending on hatcheries, and these amounts cannot be separated or subtracted to calculate only the amounts of non-hatchery restoration spending (nor do the counts of adult returning fish distinguish wild from hatchery-reared fish). Second, many conservation spending programs benefit both wild and hatchery-released fish such as predation control programs and juvenile transport. As a result, evidence that non-hatchery spending is having a positive impact on returning adult fish cannot be assumed to be affecting wild fish only. Third, the adverse density dependence effects from hatchery releases can affect both wild and hatchery fish across species and over multiple life stages. Because these confounding factors and interactions cannot be controlled to isolate the effects of conservation spending on wild fish abundance alone, we focus on evaluating their combined aggregate impacts.

We begin with a regression model to estimate the relationship between returning adult salmon and steelhead at Bonneville Dam and a set of explanatory variables. These include the ESA-related spending or investments, as well as hatchery releases and environmental factors of ocean conditions and river discharge. Our prior expectations are that recovery spending, hatchery releases and river discharge will be positively associated with adult returns [36], and that ocean conditions (PDO) will be negatively associated with adult returns [37] (although we transform the PDO variable (= 2-PDO) so that it is always positive and expected to be positively associated with adult returns). The relationship between river temperature and adult returns may be ambiguous because salmon growth and survival will generally increase with temperature up to a maximum and then decrease rapidly as temperature exceeds a physiological threshold [6]. River temperature was included in some initial model specifications, but was later dropped due to a lack of statistical significance.

The model makes it possible to predict over the period of study the levels of adult returns that are associated with both restoration spending and hatchery production. Our main model regresses adult returns on one year of restoration spending lagged by two to four years to reflect each species’ life cycle. In addition, models of cumulative spending over a period of prior years are estimated to capture evidence of multi-year impacts from durable restoration spending such as improved freshwater habitat, increasing stream complexity, or screening of irrigation canals.

Our main analysis involves two independent estimations. It employs a one-year fixed-effects spending regression model to predict the numbers of returning adult fish attributable to both spending and hatchery releases over the period of analysis. Denote that predicted level, $\hat{Z}$, which includes both hatchery adults, $\hat{H}$, and wild adults $\hat{W}$, so that $\hat{Z}=(\hat{H}+\hat{W})$. The second component of the analysis is to independently estimate annual values of the number of returning adult hatchery fish, $\hat{H}$, where $\hat{H}$ is computed by year and species as the product of the total number of released hatchery fish and their survival to adulthood (smolt-to-adult ratio, or SAR), estimated from fish tagging studies (tagging and recapturing samples of fish for statistical estimations of survival). It follows that $\hat{W}$ should be approximately equal to $\hat{Z}-\hat{H}$, so that $\hat{Z}-\hat{H}>0$ would indicate evidence of increased wild fish abundance due to conservation spending. Our null hypothesis is that $H_{0}:\hat{Z}-\hat{H}\leq 0$, or that spending does not benefit wild fish.

In a second analysis, we compare the fitted or predicted levels of adult returns over the period of study for the one-year model to the comparable predicted levels for models estimated with cumulative spending over various numbers of prior years. We expect that the predicted impacts will likely be higher for models of cumulative spending to the extent that conservation investments such as habitat restoration projects, riparian revegetation, or canal screening, will generate durable, multi-year benefits. If restoration spending makes durable improvements in freshwater habitat each year over several years, and each of these actions give rise to increases in returning adult wild fish over a period of years, then the benefits should be cumulative. In the case of hatchery releases and related spending, as well as predation control actions and fish transport, these are unlikely to have cumulative effects across multiple years or generations. Most hatchery spending involves the variable costs of producing a particular brood of releases for a single year; long-term investments in hatchery infrastructure are likely to be too diffuse to be detectable aside from their association with numbers of released fish (included separately in the model). Identification of a positive and increasing effect of cumulative spending on adult returns would lend support to a positive impact of spending on wild fish abundance.

### Data

The data and sources for the empirical model are as follows: Expenditures on ESA listed species recovery efforts come from “Federal and State Endangered and Threatened Species Expenditures, Annual Reports” [7]. These include spending by all federal agencies as well as state-spending. Annual amounts are recorded by species and basin/population, and were available from 1996–2017. For the period 1978 to 1995, data were provided by the Northwest Power and Conservation Council (but are not species-specific). All values were adjusted to 2020 dollars using the national Consumer Price Index (Fig 2). Additional relatively smaller amounts of restoration spending by NGOs and Tribes are not included. As indicated above, the spending data reported by the USFWS include some expenditures for hatchery operations and investments, whereas the hatchery release data include all hatchery releases above Bonneville Dam. We were able to identify and remove a fraction of these hatchery-related expenditures in the case of the BPA to reduce the overlap. But this was not possible for other federal and state agencies. As a result, the two variables are directly confounded.

We considered three environmental variables that influence survival: river discharge/flow, water temperature, and ocean conditions. River flow is the flow (thousands of cubic feet per second, kcfs) measured at Bonneville Dam for the period March 1 through June 30 each year. These data come courtesy of Columbia Basin Research’s Data Access in Real Time (DART)(https://www.cbr.washington.edu/dart).

For ocean conditions we use the mean annual PDO (dimensionless) from 1970 through 2020. The raw data come from NOAA’s National Centers for Environmental Information. In order to have a variable that when log-transformed (Ln) is expected to be monotonic in its relationship with ocean survival, we define the variable Ocean Conditions = 2-PDO.

River temperature is the mean daily water temperature (Celsius) measured for the period March 1 through June 30. In several years, though, temperature measurements at Bonneville Dam were missing. Therefore, to construct a complete time series for mid-basin water temperature we computed an interpolated value using data from four dams as follows: i) took daily temperature records for the period March 1 through June 30 from CBR’s DART website for the 4 lowermost dams: Bonneville, The Dalles, John Day, and McNary; ii) fit a single, biased random walk, observed with error, for each year based on the temp records for all 4 dams; iii) and calculated the mean of the estimated daily states to represent the temp index for the lower river. Note that these data come courtesy of Chris Van Holmes, as part of Columbia Basin Research’s Data Access in Real Time (DART).

Hatchery release data were obtained from the Regional Mark Information System of the Regional Mark Processing Center where all hatchery releases are available from 1965 by species, release year, subbasin, and mark type (see Fig 3). Summary statistics for the data are shown in Table 1.

**Table 1:** Descriptive statistics (includes years 1970–2020).

					
	Obs.	Mean	Std. dev.	Min	Max
ESA-related spending (million $2020/year)	197	166	150	0	453
Adult returns per year	204	227,057	224,537	8401	1,337,101
Chinook	51	468,742	266,865	185665	1,337,101
Coho	51	69,078	59,687	8401	279,717
Sockeye	51	122,861	135,810	8774	614,179
Steelhead	51	247,546	122,186	77319	633,073
Ocean conditions (PDO avg)	204	(0.26)	0.85	-2	1.32
River discharge (kcfs)	204	249.3	65.1	128	392.8
Hatchery releases/year	204	16,501,507	21,996,978	0	69,566,384
Chinook	51	50,216,176	18,909,475	3650922	69,566,384
Coho	51	6,631,716	2,220,749	654176	10,729,835
Sockeye	51	539,204	826,836	0	3,899,652
Steelhead	51	8,618,932	4,390,248	0	13,821,032

To characterize how life-cycle survival and adult return numbers are influenced by habitat or environmental variables, it is necessary to introduce lags for the years between when those influences occur and the year of return for adults in each cohort. We introduced lags in our model to reflect timing of the life-stages corresponding to the influences at the stage of juvenile emergence, out migration, ocean life-stage, and return timing as adults, as well as for expected impacts from program expenditures and hatchery releases. We introduced lags that approximate these life-stages for the influences of each factor in Table 2.

**Table 2:** Explanatory variable lags for adult returns at Bonneville Dam.

	Chinook salmon	Coho salmon	Sockeye salmon	Steelhead
Ocean conditions (PDO)*	t-2, t-3	t-1	t-2, t-3	t-2, t-3
River discharge rate	t-2, t-3	t-1	t-3	t-2, t-3
River temperature	t-2, t-3	t-1	t-3	t-2, t-3
hatchery releases	t-2, t-3	t-1	t-3	t-2, t-3
Annual expenditures	t-4	t-2	t-3	t-2
Spawner to spawner	t-4	t-3	t-4	t-4

* When two lags are indicated, their values are averaged.

### Model and methods

We measure the impacts of recovery expenditures and hatchery releases on adult returns, conditional on ocean conditions, and river flow, by estimating the following regression model where all covariates are log transformed:

$$\begin{array}{l} Returns_{it}=\beta_{0}+\beta_{1}Expenditures_{t-\tau_{i}}+\beta_{2}OceanConditions_{t-\gamma_{i}}+\beta_{3}WaterFlow_{t-\delta_{i}}+\\ \beta_{4}HatcheryReleases_{t-\theta_{i}}+\beta_{5}Spawners_{t-\varphi_{i}}+\alpha_{i}+\varepsilon_{it} \end{array}$$ (1)

where *i* indexes species and *t* indexes years. Here, *Returns_it_* is the number of fish of species *i* (chinook, coho, sockeye, and steelhead) returning at Bonneville Dam in year *t*, ${OceanConditions}_{t-\gamma_{i}}$ represents lagged Pacific Decadal Oscillation (PDO), ${WaterFlow}_{t-\delta_{i}}$ is lagged total river flow, ${HatcheryReleases}_{t-\theta_{i}}$ are lagged total fish released by hatcheries in the basin, and ${Spawners}_{t-\varphi_{i}}$ is a lagged dependent variable representing the number of spawners one generation earlier. In addition to this natural log version, the model is also estimated for the variable levels. Previous versions of the model also included mean annual water temperature in the lower river. However, there was a lack of statistical significance possibly due, as mentioned above, to the ambiguous relationship between river temperature and the fishes’ growth and survival, or to very little variation in mean river temperature over time (i.e., mean = 10.8°C, CV = 5.8%). Hence, we omit it from the final version of the model.

The main variable of interest, ${Expenditures}_{t-\tau_{i}}$, measures lagged total state and federal government expenditures on all four species. We use two alternative specifications for this variable. The first measures only one year of expenditures, and the second one measures expenditures accumulated over two, four, six, or eight years. Each variable enters the model with a different species-specific lag to reflect the correct timing of impacts on returns given differing life cycles for each species as indicated in Table 2. Finally, *α_i_* controls for time-invariant unobserved species characteristics, and *ε_it_* is an idiosyncratic error term. Standard errors are clustered at the species level. Since there are only four species, the number of clusters is small and standard errors could be underestimated. To mitigate this concern, we use a wild-bootstrap-t-method to conduct inference adjusted to the small number of clusters. The method is based on generating many bootstrap samples that resemble the actual sample (we use 999), calculating the *t–*statistic for each, and establishing how extreme the original *t*–statistic is by comparing it with the distribution of the bootstrapped statistics. The procedure does not generate standard errors, so inference is based on *p* – values [38–40].

### Estimates of hatchery adult returns

We obtain an independent estimate of the annual returning adult hatchery fish based on data for the period 2000 to 2016. The estimate is the product by year and species of a) the number of released hatchery fish and b) the associated SARs. As noted above, the number of hatchery releases come from the Regional Mark Processing Center from 1965 to the present by species, release year, subbasin, and mark type. SARs for hatchery reared fish are estimated in many tagged-fish studies across the basin each year. These studies, available from the Fish Passage Center, have produced more than 1,500 SARs estimates from 2000 to 2016. Drawing on SAR estimates with Bonneville Dam as their endpoint, the mean values from 2000 to 2016 were 0.98% for Chinook, 2.09% for Sockeye, 2.00% for steelhead, and 0.43% for Coho. The weighted average is 1.2%. The sum of the products of these two variables (hatchery releases and mean SARs for each species and release year), produce estimated returning adult hatchery salmon and steelhead at Bonneville Dam that averaged 782,000 (and an estimated 571,000 non-hatchery returning adults). The comparisons with the regression model are interpreted below.

### Results and interpretation

The estimated coefficients indicate that federal and state government expenditures are positively correlated with fish return counts (Table 3). A 1% increase in spending in a year is associated with a 0.075% in returns. Changes in cumulative spending have similar impacts. A 1% increase in expenditures accumulated over two, four, six, and eight years is associated with 0.066%, 0.059%, 0.054%, and 0.052% higher returns, respectively. The model omitting spawners produces an estimated response of 0.097%. However, additional previous spending accumulated over more than 8 years no longer has a statistically significant correlation with fish returns. Coefficients for PDO (transformed as ln(2−*PDO*)) suggest a positive relationship between favorable ocean conditions and fish counts. Evidence of a positive relationship for hatchery releases (separate from the influence of spending on hatchery programs) was not statistically significant. This appears to be due, at least in part, to the confounding effect of the inclusion of spending on hatcheries in the restoration spending variable. When spending and hatchery releases were jointly tested for significance, they were found to be statistically significant at the 1% levels for models 1–4 and at the 5% level for model 5. The estimated coefficient on spawners of 0.39 to 0.4 is significant for all versions of the model shown in Table 3. This coefficient can be interpreted as an elasticity, or ratio of percent changes of adult returns in response to a percent change in spawners. Over time with a stable population of naturally spawning fish we might expect the value of this elasticity to be near 1.0. Given the absence of any spawner-to-spawner relationship for hatchery fish, a coefficient of 0.4 would be consistent with populations of salmon and steelhead that over the period of study average 40% wild and 60% hatchery fish. Indeed, the SAR-based estimate of hatchery adult returns averages 58% of total adult returns over the period 2003–2018.

**Table 3:** Regression results for panel models estimating adult returns at Bonneville Dam.

Model #	(1)	(2)	(3)	(4)	(5)
Variable	One-year spending^(a)^	2 yr. cum. spending^(a)^	4 yr. cum. spending^(a)^	6 yr. cum. spending^(a)^	One-year spending, no spawners^(a)^
Ln(Total spending)	0.0747**				0.0974**
	(0.0198)				(0.00953)
Ln(Hatchery releases)	0.00965	0.00998	0.0100	0.00594	0.0184*
	(0.0202)	(0.0203)	(0.0203)	(0.0221)	(0.0131)
Ln(2–Pacific Decadal Oscillations)	1.092**	1.096**	1.089**	1.108**	1.354**
	(0.241)	(0.240)	(0.235)	(0.256)	(0.324)
Ln(River flow)	0.321	0.319	0.316	0.393	0.230
	(0.182)	(0.184)	(0.183)	(0.206)	(0.193)
Ln(Spawners)	0.394*	0.395*	0.396*	0.399*	
	(0.0806)	(0.0800)	(0.0799)	(0.0831)	
Ln(Total spending (2-yrs cum.)		0.0658***			
		(0.0168)			
Ln(Total spending (4-yrs cum.)			0.0592**		
			(0.0150)		
Ln(Total spending (6-yrs cum.)				0.0537**	
				(0.0142)	
Observations	187	187	187	182	192
R-squared	0.756	0.755	0.755	0.755	0.700

Results for model in (1) in logs. Column 1 shows estimates for total spending measured only for one (lagged) year, and columns 2–4 show estimates for cumulative spending over two, four, and six lagged years. Column 5 modifies the model in (1) by omitting spawners. Species-level cluster-robust standard errors are in parentheses. Significance of *p—*values are indicated by: *** *p* < 0.01, ** *p* < 0.05, * *p* < 0.1.

^(a)^ Spending and hatchery releases jointly significant at the 1% level in Eqs 1–3 and the 5% level in model 4. R-squared values indicate the models explain between 70% and 76% of variation in annual adult returns.

The model estimates for variable levels in Table 4 are similar, if slightly less significant in terms of goodness of fit and in terms of the significance of some individual variables compared to the log-transformed model. One advantage of the levels model is its direct interpretation of the coefficients. A $1 million increase in spending in a year over the period of study was associated with an incremental increase of 374 adult returning fish (per species, or 1496 for four species). Caution is warranted against extrapolation and prediction outside of sample based on these estimates due to several factors. These include the nonlinear effects of density dependence, and also the fact that the specific past actions introduced as spending increased from zero to $400 million per year cannot be assumed to be repeatable with similar responses in most cases; additional spending would necessarily involve actions with impacts that would differ from past interventions.

**Table 4:** Regression results for panel models estimating adult returns at Bonneville Dam.

Model #	(6)	(7)	(8)	(9)	(10)
Variable	One-year spending	2 yr. cum. spending	4 yr. cum. spending	6 yr. cum. spending	One-year spending, no spawners
Total Spending	374.2*				442.9**
	(180.6)				(160.9)
Hatchery Releases	0.00186	0.00202	0.00191	0.00151	0.00178**
	(0.000524)	(0.000607)	(0.000607)	(0.000841)	(0.000509)
Ocean conditions (2–Pacific Decadal Oscillation)	88,860*	89,130*	86,596*	88,964*	87,115
	(46,469)	(45,204)	(43,663)	(42,675)	(45,638)
River flow	232.2	214.2	191.8	271.1	213.2
	(163.9)	(163.9)	(150.2)	(175.4)	(170.2)
Spawners	0.0885	0.0863	0.0770*	0.0787	
	(0.0943)	(0.105)	(0.105)	(0.108)	
Cumulative spending (2 yrs.)		184.8*			
		(106.3)			
Cumulative spending (4 yrs.)			101.3*		
			(56.60)		
Cumulative spending (6 yrs.)				65.36*	
				(40.31)	
Observations	188	187	187	182	193
R-squared	0.650	0.645	0.648	0.650	0.650

Results for model in (1) in levels. Column 6 shows estimates for total spending measured only for one (lagged) year, and columns 7–9 show estimates for cumulative spending over two, four, and six lagged years. Column 10 modifies the model in (1) by omitting spawners. Species-level cluster-robust standard errors are in parentheses. Significance of *p—*values are indicated by: *** *p* < 0.01, ** *p* < 0.05, * *p* < 0.1. R-squared values indicate the models explain about 65% of variation in annual adult returns.

In the two-year cumulative model that value is 185 for an increase in a year, but because of the model’s structure, an increase by $1 million in one year must necessarily involve an identical increase in the following year. As a result, we recognize that the annual marginal effect represented by the coefficient produces a total effect over two years of double that amount, or an additional 370 adult returning fish. Applying the same reasoning for the 4-year and 6-year models produces total effects for a $1 million increase of 405 and 392 additional adult returning fish, respectively. Taken together the total effects appear to vary little between the one-year model and the cumulative spending models.

**Fig 4:**
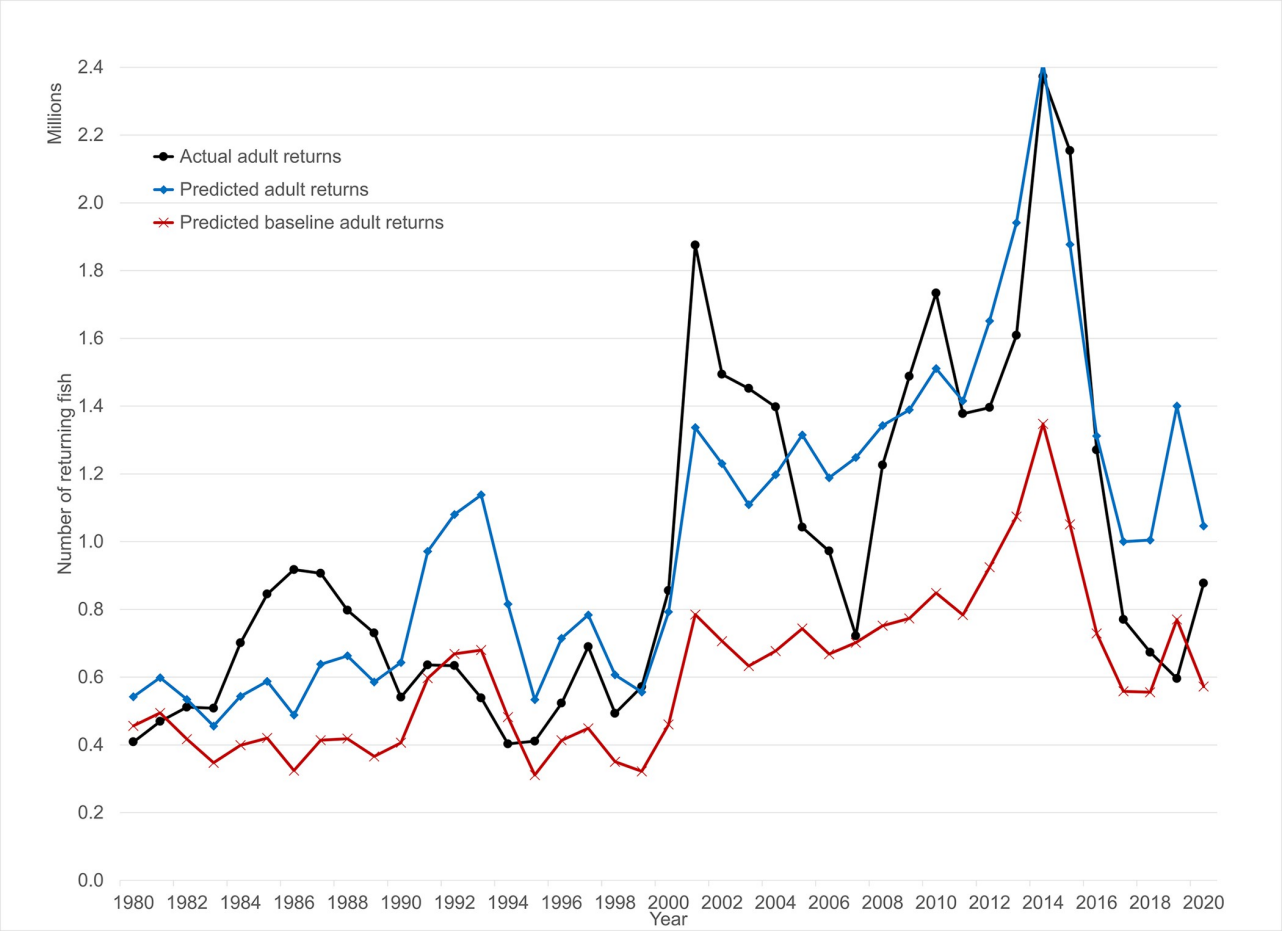
Actual and model-fitted (model 1 Table 3) adult returns at Bonneville Dam.

These regression models can be used to generate predicted values for adult returns for each year over the period of analysis based on the values for the explanatory variables and the estimated coefficients. However, when the dependent variable in a regression is log-transformed, ln(y), the obvious inverse transformation we use to get fitted values systematically underestimates the expected value of y, or in other words is a biased estimator of the mean of y [41]. To minimize this bias, a method proposed in the literature uses the standard error of the regression to generate adjusted results. These predictions of adult returns are close to observed adult returns (Fig 4). The estimates of adult returns can be further decomposed to delineate the contributions of individual explanatory variables toward the overall level of adult returns in a given year or over time. For example, the model indicates a strong positive effect of ocean conditions in 2002 and 2014.

Versions of these models were also run with a time trend or year variable to test for evidence of a trend over the period of analysis (e.g., might adult returns have declined were it not for spending and/or hatchery releases?). In the case of the log-transformed models, no trend is detected (the estimated coefficient is zero). For the levels models the trend coefficient is slightly positive but not significant.

The fitted results can also be used to predict the level of adult returns in a counterfactual situation, for example one without expenditures or hatchery releases. In the case where expenditures and hatchery releases (or their logs) are set to zero, predicted returns can be calculated to reflect a “baseline” of adult returns as shown in Fig 4.

Due to the multiple confounding factors involving restoration spending and hatchery releases, as discussed previously, their estimated coefficients are likely to be biased in a way that prevents direct inferences about spending impacts on wild fish. This compels us to take an indirect approach.

### Evidence of impacts above hatchery impacts

**Fig 5:**
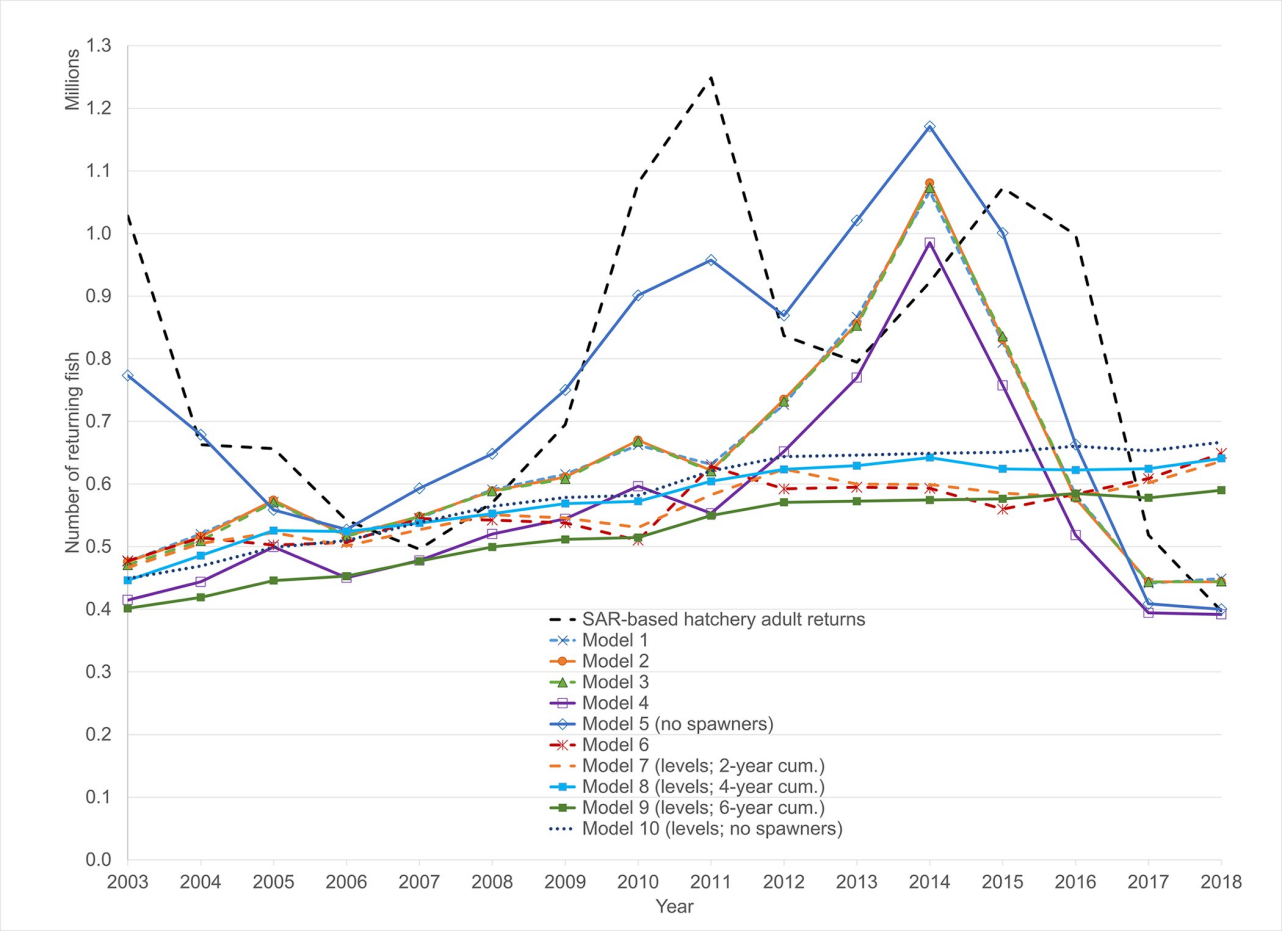
Predicted adult returns due to both spending and hatchery releases (various model specifications), and compared to SAR-based estimate of hatchery adult returns.

First, based upon the one-year empirical model, the predicted composition of adult returns due to both spending and hatchery releases, $\hat{Z}=(\hat{H}+\hat{W})$, averaged 631,000 fish per year from 2003–2018 (Fig 4). Second, the estimated hatchery adult returns, computed as the product of the hatchery released fish and their annual survival rates (SARs), averaged 782,000 over the period (see Fig 5). The null hypothesis of no impact of conservation spending on wild salmon and steelhead can be represented as H_0_: $\hat{Z}-\hat{H}\leq 0$, or that the model estimate of adult returns associated with spending and hatchery releases is less than or equal to the independently estimated hatchery adult returns.

For the period 2003–2018 for which we have both estimates, we compare our 10 models from Tables 3 and 4. For model 1, $\hat{Z}-\hat{H}$ averages -152,000, or 19% lower than $\hat{H}$, and is estimated to be above $\hat{H}$ only 5 years out of 16. In models #2 to #5, $\hat{Z}-\hat{H}$ is negative by 24%, 24%, 40%, and 5%, respectively. For models 6–10, is $\hat{Z}-\hat{H}$ is negative and below $\hat{H}$ by -21% to -51% (Fig 5). Taken together we cannot reject the null hypothesis of no impact of conservation spending on wild salmon and steelhead over this period.

### Evidence of durable impacts for wild fish

The estimation of a range of cumulative spending models serves as a robustness check on the one-year model. As indicated above, the estimated coefficients for the models in Table 4 correspond to total impacts for an incremental $1 million that vary only marginally between the 1-year model and the six-year model (between 370 and 405 adult returning fish per species). In addition, comparison of the cumulative spending models could reveal evidence of the impacts of durable investments in freshwater habitat on wild fish. We expect the models that include cumulative spending to capture the extent to which spending in a given year may generate benefits over multiple years, and that those cumulative impacts on adult returns may be a multiple of its one-year impacts. Models which included more than 6 years of spending did not produce statistically significant coefficients for spending. Overall, however, the models produced results with ‘goodness-of-fit’ very similar to the one-year model.

The predicted values for the log-transformed models are tightly clustered (models 1–5), as are the levels models (models 6–10) (Fig 5). When accounting for cumulative spending in two or more years prior to the juvenile migration year of a given cohort (and correcting as above for the bias due to having a log-transformed dependent variable), the predicted impact is essentially the same as for the one-year model. For the log-transformed models, the predicted adult returns due to both spending and hatchery releases for cumulative spending models were lower than for the one-year model. In the levels models these predictions were within 5% over the period for the one-year predictions. Overall, these comparisons do not provide supportive evidence of durable and compounding impacts of spending on wild adult returns.

## Discussion

Evidence of the benefits to salmon and steelhead of the restoration programs in the CRB has been elusive. This is due in part to the multitude of disparate projects, programs and actions diffused over space and time, as well as the basin’s large geographic scale, variability of environmental factors, and data collection challenges. The density dependence of fish survival and its compounding interactions between wild and hatchery fish, and between and among species, present particularly elusive relationships to capture empirically. These factors combine to make identifying evidence of changes in abundance attributable to or associated with the wide range of restoration programs problematic.

Our analysis takes advantage of the availability of 50 years of annual counts of adult returns at Bonneville Dam, the lowermost dam on the mainstem of the Columbia River, for a panel of four species, as well as the complementary availability of environmental variables for ocean and riverine conditions, hatchery releases, survival rates for hatchery released fish, and conservation spending. The empirical models we’ve estimated explain 65% to 75% of the variations in adult returns. The increment in R-square (squared semi-partial correlations) in the levels model (not including the categorical variables) are 2% for conservation spending, 23% for hatchery releases, 19% for ocean conditions, and 7% for spawners.

The aim of our study has been to look for evidence of the return on investment for the $9 billion restoration spending in the CRB over the past four decades. That return or impact would be expected in the form of increased “returns” of wild adult salmon and steelhead at Bonneville Dam. Our study does find evidence of changes in adult returns positively associated with contemporaneous changes in spending (lagged to reflect species life cycles), but these impacts also reflect commingling of restoration spending with spending on hatchery releases, the impacts of spending on hatchery fish survival, and the density dependence effects of hatchery releases. As a result, evidence of benefits to wild fish alone requires indirect approaches. To accomplish this, the model’s predicted adult returns (both hatchery and wild fish) attributed to both spending and hatchery releases are compared to independent estimates of returning hatchery fish based on hatchery survival estimates (smolt-to-adult ratios). The comparison finds the model-predicted levels of adult returns due to spending and hatchery releases do not exceed the survival-rate based estimates for hatcheries alone; this means we find no empirical evidence of an increase in wild fish abundance associated with restoration spending.

In a second analysis, the results from the one-year spending model were compared to models of cumulative spending across multiple years. The cumulative models could potentially provide evidence of relatively higher impacts if wild fish are benefiting from durable habitat improvements over multiple years, whereas no similar cumulative effects would be expected for hatchery fish. These comparisons also did not indicate evidence of cumulative benefits from conservation spending.
